# Exploring the potential of ChatGPT as a digital advisor in acute psychiatric crises: a feasibility study

**DOI:** 10.1007/s00115-025-01837-3

**Published:** 2025-06-06

**Authors:** Lubomir Barabas, Michal Novotny, Dennis Jung, Thomas Müller, Nicolas Nagysomkuti Mertse

**Affiliations:** 1 Private Clinic for Psychiatry and Psychotherapy Meiringen, Meiringen, Switzerland; 2 conceito GmbH, Leutzenheldstraße 4, 76327 Pfinztal, Germany; 3 Translational Research Center, University Hospital of Psychiatry and Psychotherapy, University of Bern, Bern, Switzerland; 4 University Hospital of Old Age Psychiatry and Psychotherapy, University of Bern, Murtenstrasse 21, 3008 Bern, Switzerland

**Keywords:** Large language model, Chatbot, Psychiatric crisis management, Artificial intelligence, Digital intervention, Großes Sprachmodell, Chatbot, Management psychischer Krisen, Künstliche Intelligenz, Digitale Intervention

## Abstract

**Background:**

This exploratory study tested ChatGPT as a digital advisor chatbot for German-speaking individuals in acute psychiatric crises. Additionally, the attitudes of young physicians and psychologists towards the use of large language models (LLMs) in healthcare were investigated.

**Methods:**

In total, 20 resident physicians and psychologists simulated patients in three clinical scenarios (depression, psychosis, adjustment disorder) and interacted with ChatGPT. They evaluated the chatbot’s performance regarding overall experience, pleasantness, appropriateness of the responses, realism, and helpfulness. Before and after the intervention, their attitudes towards such a chatbot were assessed. Finally, they assessed 12 statements about the future of LLMs in healthcare and provided open feedback on the chat experience.

**Results:**

ChatGPT received predominantly positive ratings (over 8/10 points) for overall experience, helpfulness, pleasantness, and appropriateness, while realism was rated slightly lower at 7/10 points. The appropriateness of the responses varied significantly between the scenarios, with lower ratings for the psychosis scenario. Open feedback confirmed the limited suitability of ChatGPT for psychosis patients. Overall, 70% or more of the participants agreed that LLMs will become increasingly important in everyday life and healthcare, and that an LLM-based chatbot would be a modern tool for low-threshold access to initial psychiatric aid. However, the high number of neutral responses across all 12 items (20–45%) indicates uncertainty regarding the actual benefits and risks.

**Conclusion:**

The performance of ChatGPT was rated positively overall by the participants. Significant practical and methodological limitations remain, however, highlighting the need for further research including real patients for a gradual, carefully monitored integration of LLMs into mental healthcare.

**Supplementary Information:**

The online version of this article (10.1007/s00115-025-01837-3) contains supplementary material, which is available to authorized users.

## Abstract (de)

**Hintergrund:**

In dieser explorativen Studie testeten wir ChatGPT als digitalen Berater für deutschsprachige Personen in akuten psychischen Krisen. Zusätzlich wurde die Einstellung von jungen Ärzt:innen und Psycholog:innen zur Nutzung von „large language models“ (LLM) im Gesundheitswesen untersucht.

**Methoden:**

Dazu simulierten 20 Assistenzärzt:innen und Psycholog:innen Patient:innen in 3 Krisenszenarien (Depression, Psychose, Anpassungsstörung) und interagierten mit ChatGPT. Anschließend bewerteten sie die Leistung des Chatbots hinsichtlich Gesamterlebnis, Nutzen, Annehmlichkeit, Realismus und Angemessenheit der Antworten. Abschließend bewerteten sie 12 Aussagen zur Zukunft von LLM im Gesundheitswesen und gaben ein offenes Feedback zum Chaterlebnis.

**Ergebnisse:**

ChatGPT erhielt überwiegend positive Bewertungen (über 8/10 Punkte) für Gesamterlebnis, Nutzen, Annehmlichkeit und Angemessenheit, wobei der Realismus mit 7/10 Punkten etwas niedriger bewertet wurde. Die Angemessenheit der Antworten variierte signifikant zwischen den Szenarien, mit niedrigeren Bewertungen für das Psychoseszenario. Das offene Feedback bestätigte die begrenzte Eignung für Psychosepatienten. Von den Teilnehmenden stimmten 70 % und mehr zu, dass LLM im Alltag und Gesundheitswesen an Bedeutung gewinnen werden und ein LLM-basierter Chatbot ein zeitgemäßes Instrument für einen niederschwelligen Zugang zur psychiatrischen Ersthilfe wäre. Die hohe Anzahl neutraler Rückmeldungen bei allen 12 Items (20–45 %) weisen jedoch auf Unsicherheit bezüglich der tatsächlichen Vorteile und Risiken hin.

**Schlussfolgerung:**

Die Leistung von ChatGPT wurde von den Teilnehmenden insgesamt positiv bewertet. Es bestehen jedoch signifikante praktische und methodische Einschränkungen, die die Notwendigkeit weiterer Forschung unter Einbeziehung realer Patient:innen für eine schrittweise sorgfältig überwachte Integration von LLMs in die psychische Gesundheitsversorgung hervorheben.

## Background

The rapid advancement of large language models (LLMs) has demonstrated their potential to revolutionize various sectors including medicine. Since the public release of ChatGPT in November 2022, numerous papers have discussed the potential uses in psychiatry [23, 26]. In this study, our *first aim* was to test ChatGPT as a digital advisor in the context of acute psychiatric crises. Individuals experiencing such crises often face significant challenges in accessing timely and appropriate mental health support [17]. The scarcity of resources and the stigma surrounding mental illness can exacerbate their distress, leading to delayed search for help and hence to adverse outcomes [19]. Chatbots based on LLMs offer a potential avenue for providing immediate support during these vulnerable moments. A major advantage of LLMs is their ability to converse in numerous languages, which is quite relevant in view of multilingualism and the high migrant rate both in Switzerland and Germany [11, 25]. In a recent Swiss survey, 17% of the respondents stated being open to using chatbots for health information, reflecting a broader trend [1, 13].

Previous studies on outcome prediction using machine learning methods already showed promising results before the emergence of LLMs [14]. Recent advancements in natural language processing technology now enable the transformation of conversations, which form the core of psychotherapeutic diagnostics and treatment, into analyzable data, which significantly expands the range of potential uses in diagnostic and therapeutic contexts. Sadeh et al. demonstrated how the analytical capabilities of a dedicated mental health platform could positively influence the therapeutic process by providing constructive feedback for the therapist and significantly reduce the documentation effort [22].

However, the unsupervised use of LLMs in patient care, particularly during acute crises, raises significant ethical, practical, and safety considerations. While research on LLMs in mental health is still nascent, the existing literature provides a foundation for understanding both their potential and the associated challenges. Levkovich and Elyoseph (2023) found that ChatGPT’s responses demonstrated higher adherence to treatment guidelines for depression compared to those of primary care physicians (PCPs; [18]). Notably, they also reported that ChatGPT did not exhibit the same gender or socioeconomic biases observed in the responses of PCPs. Subsequent work by the same authors found that the prognoses made by ChatGPT‑4 for schizophrenia and depression demonstrated high concordance with the assessments of mental health professionals [8, 9]. However, several studies have also revealed important limitations. Heston’s findings reveal a concerning tendency for LLMs to delay human intervention in cases of user suicidality, creating a potential patient safety hazard [16]. Dergaa et al. highlighted that the quality of LLM-generated treatment recommendations for sleep disorders decreased with increasing case complexity [5]. Alanezi reported that patients using ChatGPT for mental health support expressed concerns regarding data privacy, information accuracy and reliability, and cultural and linguistic limitations [2]. Yet, some participants acknowledged its potential utility in crisis intervention, which underscores the necessity for further investigations into the efficacy and safety of artificial intelligence (AI)-driven crisis intervention in mental healthcare.

Our *second aim* was to assess the attitude of young psychiatrists and psychologists towards first-responder chatbots and AI in mental healthcare—an important demographic, as the successful implementation also hinges on the perceptions of these future clinicians. Previous research on medical professionals’ attitudes towards AI revealed a spectrum of optimism and caution. Ghadiri et al. highlighted the perceived utility of AI among PCPs in adolescent mental healthcare, particularly for data analysis, while also raising concerns regarding potential clinical skill atrophy and ethical dilemmas [12]. Moldt et al. investigated medical students’ perceptions of medical chatbots, demonstrating a generally positive outlook tempered by concerns about data privacy and over-reliance, emphasizing the need for structured AI and data literacy education within medical curricula [20]. Blease et al. examined psychiatrists’ experiences with generative AI, revealing its utility for administrative tasks but also underscoring ethical considerations [4]. These studies collectively indicate a growing interest in the potential of AI in healthcare, alongside a shared awareness of the ethical, privacy, and educational challenges that ought to be addressed for a successful integration.

## Methods

### Study population and design

This exploratory study employing a semi-experimental design was conducted at the Psychiatric Clinic of Meiringen between July and September 2023. To mitigate potential risks to patient safety and privacy, and to reduce the influence of patient variability on the evaluation, we recruited resident physicians and psychologists from the clinic to simulate patient interactions. Participants were enrolled on a voluntary basis with no other specific inclusion or exclusion criteria. The study was granted ethical exemption by the Cantonal Ethics Committee of Bern (BASEC-Nr: Req-2023-00774) and all participants provided informed consent prior to participation. As compensation for their time, participants received a complimentary meal at the clinic’s restaurant. The total survey duration was estimated to be 20–30 min, including an optional 5‑min break.

### Assessment of ChatGPT’s performance

ChatGPT (version 3.5) was prompted to simulate a psychiatric first-responder chatbot. Participants were randomly assigned one of three clinical scenarios (psychosis, depression, adjustment disorder) developed for this intervention. They were then instructed to role-play the patient described in their assigned scenario and engage in a chat with ChatGPT to obtain guidance on their presented problem. Participants were given autonomy to conduct the chat as they deemed appropriate and to conclude the conversation at their discretion.

ChatGPT’s performance was then assessed using a 10-point Likert scale rating the overall experience, the pleasantness of the chat, the appropriateness of the responses, the realism of the chatbot, and the helpfulness of the advice. To compare the performance of ChatGPT with the performance of pre-LLM chatbots, the participants were also asked to rate their prior experiences with customer service chatbots on similar scales before starting the chat.

### Assessment of the attitude towards an advisor chatbot

To take in account the impact of the chatting experience and to objectify the impact of personal experience on the attitude of participants, we assessed the attitude both before and after the chat with ChatGPT with the following statements to be answered with “yes”/“no”/“don’t know”:

1. It would be used by patients. (Hypothesized patients’ subjective norm)
2. It would be recommended by outpatient psychiatrists (Subjective norm)
3. It would relieve the psychiatric emergency department. (Anticipated benefit)
4. It would be helpful for patients in a crisis situation. (Expectancy of usefulness)
5. It would integrate well into the emergency consultation process. (Compatibility)

### Expectations and attitudes regarding the use of LLMs in healthcare and psychiatry

The survey concluded with the rating of 12 statements regarding the general attitude and future expectations of the participants towards the use of LLMs in healthcare and psychiatry (Fig. 3). The items were developed iteratively by the research team following a review of the relevant literature and were designed to capture a range of optimistic and pessimistic viewpoints.

### Statistical analysis

**Fig. 1:**
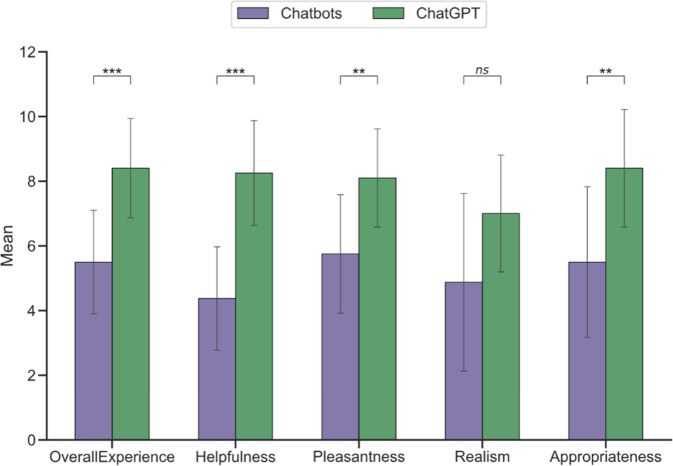
Performance of previous contacts with chatbots (purple) vs. performance of ChatGPT (green). *ns* not significant, *p > 0.05, **p ≤ 0.01, ***p ≤ 0.001

**Fig. 2:**
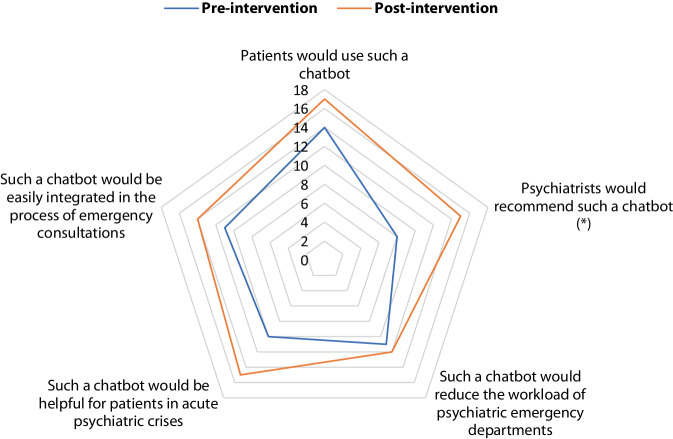
Spider chart of the participants’ attitude towards a chatbot advising patients in acute psychiatric crises. Pre-intervention “yes” answers are shown in blue, post-intervention “yes” answers in orange, other options (“no”/“don’t know”) are not included in the chart. Statistically significant differences (*p* < 0.05) are marked with (*)

Unpaired *t* tests were performed for the comparison of previous chatbot experiences versus ChatGPT and are displayed in Fig. 1. The attitude towards chatbot advising pre- and post-intervention displayed in Fig. 2 was evaluated using Fisher’s exact test with a 2 × 3 contingency table. An analysis of variance (ANOVA) was conducted on the dataset to examine the differences between the clinical scenarios. Due to heterogeneity in variances, Welch ANOVA was implemented followed by the Games–Howell post hoc test in the case of significance (Fig. 2). The assumption of normal distribution of the data was validated by visual inspection of the residuals.

All computations were performed with Python (3.10.12). Graphics were generated with Seaborn (0.13.1) and Matplotlib (3.8.0). Pandas (1.5.2) and Numpy (1.26.3) were used for ordinary data steps. Statistical analyses were performed with Pingouin (0.5.4). All libraries were set up in a Conda (4.11) environment. Python scripts can be found in the supplementary data (python_scripts.zip). The full result output can be found in the supplementary material (statistics.xlsx).

## Results

### Population

In total, 20 participants were enrolled in the study, including 7 psychologists and 13 resident physicians (Table 1). All participants completed the whole survey; one participant had to interrupt the chat prematurely due to server problems.

**Table 1:** Study population characteristics

	Male	Female
Total	5	15
Age	(26–30 years) 4; (31–35 years) 1	(26–30 years) 1; (31–35 years) 8; (36–40 years) 5; (> 40 years) 1
Years of training	2.6 years (range: 1–3 years)	2.4 years (range: 1–7 years)
Swiss nationality/other nationality (EU)	0/5	7/8
Psychiatry/other specialty/psychologist	5/0/0	4/4/7

### Evaluation of ChatGPT’s performance as a psychiatric advisor chatbot

The overall experience of the intervention was rated as positive with a mean of 8.4/10 (± 1.49). The counselling was rated as helpful with a mean of 8.25/10 (± 1.58). The interview was rated as pleasant with a mean of 8.1/10 (± 1.48). The realism was rated as rather good with a mean of 7/10 (± 1.76). The appropriateness of the responses was rated as good with a mean of 8.4/10 (± 1.77). Respondents with a psychosis scenario rated the advice as significantly less appropriate than other respondents: mean depression scenario, 8.4/10 (± 2.9); mean adjustment disorder scenario, 9/10 (± 0.8); mean psychosis scenario, 7.7/10 (± 0.5); *p* = 0.019.

Compared to previous experience with chatbots, the advice provided by ChatGPT was rated as significantly better in all dimensions except realism (see network diagram in Fig. 1).

### Attitude towards chatbots as first responders for patients in acute psychiatric crises and impact of personal experience

The results of the attitude assessment are presented in Fig. 2. Pre-intervention/post-intervention comparison showed a significant improvement in participants’ expectations following the intervention in terms of recommendations by psychiatrists. There was also a trend towards better usefulness for patients in acute psychiatric crisis as well as towards higher expected use and better integration into the emergency consultation process. There was no relevant difference in the pre/post comparison in terms of reducing the burden on the psychiatric emergency department.

### Expectations and attitudes regarding the use of chatbots in healthcare and psychiatry

**Fig. 3:**
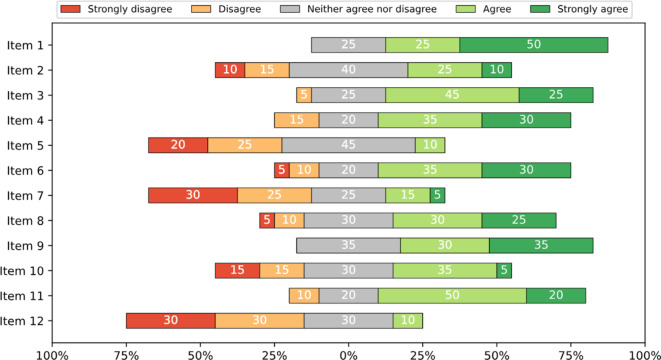
Future expectations regarding large language models (LLMs) rated on a 5-point Likert-scale (1 = I do not agree;5 = I completely agree). Items: (1) I believe that LLMs will become more important in everyday life. (2) I believe that the potential future benefits of such LLMs in healthcare are overestimated. (3) I believe that such LLMs will fundamentally change everyday working life in the healthcare sector. (4) I believe that the use of LLMs in healthcare makes sense. (5) I consider the use of such LLMs in the healthcare sector to be dangerous. (6) I think the use of such LLMs in psychiatry makes sense. (7) I consider the use of LLMs in psychiatry to be dangerous. (8) The use of such LLMs should be considered for psychiatry in the future. (9) A first-responder chatbot based on such LLMs would represent a straightforward way of establishing low-threshold contact with psychiatry in the case of need. (10) The use of LLMs would be questionable in terms of data privacy. (11) The use of such LLMs would be a modern tool in the management of psychiatric crises. (12) I think that the use of such LLMs in healthcare will have a negative impact on patient care

The participants’ expectations are shown in Fig. 3. A clear majority (70% and more) agreed that LLMs are likely to become more important in the future, will fundamentally change everyday work in healthcare, and would be an appropriate timely tool for managing acute psychiatric crises. A majority (50% and more) also agreed that the use of LLMs in healthcare in general and in psychiatry in particular would be useful, would provide a low-threshold contact option, and should therefore be considered for use in psychiatry. Over 50% disagreed that the use of LLMs in psychiatry would be dangerous and that the impact of their use on patient care would be negative. There were divergent opinions on whether the benefits of LLMs were overestimated and regarding concerns about data privacy.

### Personal feedback

Five participants provided personal feedback. Two out of five participants with a psychosis scenario stated that the chatbot was rather not suitable for their specific scenario, as paranoia may impede trust in the chatbot’s advice, but that it could certainly be useful for patients with affective disorders. The overall feedback was rather positive, mentioning the surprising complexity and empathy (one participant), and emphasizing the good professional quality of the advice (two participants).

## Discussion

The positive reception of ChatGPT as psychiatric chatbot, particularly its perceived helpfulness, pleasantness, and appropriateness, aligns with studies hinting at the potential of LLMs in delivering mental health interventions and support [2, 10, 21]. The authors of last year’s Swiss e‑health barometer emphasized the need for a low-threshold point of access for mental health support services in the conclusion of their study, as a large part of the Swiss population seems to struggle in finding reliable information on mental health topics [13]. An earlier referral to a psychiatrist or general practitioner, as a potential result of this low-threshold setting, could reduce the severity of the disorder at the start of treatment, thereby reducing both psychiatric emergency admissions and the burden of inpatient hospitalization [6].

However, the variation in the perceived appropriateness of ChatGPT’s responses across different clinical scenarios hints at the challenges in applying LLMs to complex mental health conditions. This aligns with concerns raised by Elyoseph and Levkovich and Dergaa et al., who warn about relying solely on LLM-generated assessments in cases where complex clinical judgment is required [5, 7]. The reliance of LLMs on text-based pattern recognition indeed poses a substantial risk of delivering inappropriate and potentially harmful advice, particularly during mental health crises. This is acutely concerning in cases of suicidal ideation, where misinterpretations may lead to dangerous recommendations [15]. Bartal et al. showed that training the chatbot with relevant high-quality data can significantly improve the output [3]. Feeding the chatbot with data including general diagnostic pathways and psychiatric red flags such as suicidality might tailor recommendations. Another strategy to improve the reliability of LLM-based therapeutic communication could involve a review before transmission by tele-therapists. Although less time-efficient than direct LLM–patient interactions, this process ensures quality control over the LLM responses. However, other important practical and ethical considerations remain and should be addressed.

Primarily, as text-based systems, LLMs cannot interpret crucial nonverbal cues and they lack cultural sensitivity, which is essential for a comprehensive mental health assessment [2]. Secondly, research shows that users tend to over-rely on the accuracy of information delivered by LLMs, which may discourage patients from seeking timely professional mental health assistance rather than facilitating it [5, 24]. Thirdly, technical challenges, such as AI hallucination and insufficient technological literacy among users or connectivity, further compromise the accessibility and reliability of these systems. Fourthly, given the limited regulation of AI, the storage and potential use of sensitive mental health data for LLM training pose significant data privacy concerns, underscoring the need for robust legal and ethical frameworks. Finally, LLMs lack real-time safety protocols, such as direct emergency service contact, presenting a critical limitation when users are in immediate danger. These limitations should be addressed prior to the integration of LLMs in mental healthcare.

### Expectations and attitudes regarding the use of LLMs in healthcare and psychiatry

Participants acknowledged the growing relevance of LLMs, expressing optimism about their future role in daily life and healthcare. However, when specifically considering the benefits and risks within psychiatric care, a more nuanced perspective arose. While recognizing potential advantages, particularly in psychiatric first aid, the participants voiced data privacy concerns, hinting at concerns regarding the secure storage and potential misuse of sensitive patient information. This aligns with findings from the studies by Blease et al. and Moldt et al., where psychiatrists and medical students expressed similar concerns.

Notably, a high frequency of neutral responses, especially for items 2 and 3 (overestimated benefits and fundamental change), suggests a prevailing uncertainty regarding the transformative potential of LLMs. This indicates that participants are not yet fully convinced of the full extent of the benefits and maintain a keen awareness of the associated risks. This underscores the need for continued research and open discourse on the future integration of LLMs within mental healthcare.

### Methodological limitations

Our study has several methodological limitations that warrant consideration. First, we employed a quasi-experimental design with a rather small sample size limited to junior doctors and psychologists with limited clinical experience at a single institution, not actual patients in crisis. Therefore, the findings may be biased in favor of LLMs and not accurately reflect how patients from diverse backgrounds with varying levels of technological literacy would handle the chatbot in real-world scenarios. A randomized controlled trial with real patients comparing ChatGPT with face-to-face or telephone-based counseling may provide stronger evidence of the chatbot’s efficacy relative to other interventions.

Second, the use of predefined clinical scenarios does not fully capture the complexities of real-life mental health crises and challenges that could arise in genuine crisis situations.

Third, the rigid questionnaire used in the study limits the depth and breadth of responses, whereas a semi-structured interview may have been more effective in capturing nuanced insights.

Fourth, the survey assessing future expectations regarding the use of LLMs in healthcare was conducted after participants were exposed to ChatGPT, raising concerns about potential bias in responses and the transferability of the findings.

Fifth, the absence of focus group interviews or a comparable qualitative approach in item generation may have resulted in the omission of key attitudinal dimensions.

Finally, the study employed the free version of ChatGPT, which was not state of the art then and is not state of the art now. While it was assumed that a more advanced model would perform better, this remains unproven.

## Conclusion

While large language models (LLMs) demonstrate potential as accessible mental health support tools, their application necessitates careful consideration of significant limitations. Our study revealed that the perceived appropriateness of ChatGPT’s responses varied across clinical scenarios, underscoring the challenges of relying on LLMs for nuanced mental health assessments. Numerous clinical limitations, especially the risk of over-reliance and misinterpretation of LLM-generated advice and the lack of real-time safety protocols, need to be addressed before implementation. The same applies for ethical and practical concerns surrounding data privacy and regulatory frameworks. Finally, the methodological limitations of our study highlight the need for robust, patient-centered research, including randomized controlled trials and qualitative analyses, to fully evaluate the efficacy and safety of LLMs in mental health settings.

The assessment of attitudes and future expectations reflected this nuanced appreciation among participants, highlighting a prevailing uncertainty regarding the transformative potential of LLMs in psychiatric care and raising concerns about data privacy. This uncertainty, coupled with the observed variability in perceived appropriateness, underscores the need for a phased and carefully monitored integration of LLMs into mental healthcare.

## Supplementary Information

Items Expectations, Clinical Scenarios (*Items zu Erwartungen, Kasuistiken*)

Statistics

ANOVA

Compare bots

Fisher main

Fisher’s exact test

Future expectations

Survey

## Data Availability

Python files and ANOVA tables can be found in the supplementary material. Other data will be made available on reasonable request.

## Declarations

The study was granted ethical exemption by the Cantonal Ethics Committee of Bern (BASEC-Nr: Req-2023-00774) and all participants provided informed consent prior to participation.
