# Supplementary material for: Exploring the potential of ChatGPT as a digital advisor in acute psychiatric crises: a feasibility study
Source: Nervenarzt. 2025 Jun 6;97(3):265–71. doi: 10.1007/s00115-025-01837-3 (PMC13171642; doi:10.1007/s00115-025-01837-3)
Supplement: Supplementary file 1 — Items Expectations, Clinical Scenarios (Items zu Erwartungen, Kasuistiken) [file 115_2025_1837_MOESM1_ESM.docx]

**Items betreffend Erwartungen an die Zukunft:**

1. Ich glaube, dass Sprachmodelle wie ChatGPT im Alltag an Bedeutung gewinnen werden.
2. Ich glaube, dass der potenzielle künftige Nutzen von solchen Sprachmodellen im Gesundheitswesen überschätzt wird.
3. Ich glaube, dass solche Sprachmodelle den Arbeitsalltag im Gesundheitswesen grundlegend verändern werden.
4. Ich halte den Einsatz von Sprachmodellen wie ChatGPT im Gesundheitswesen für sinnvoll.
5. Ich halte den Einsatz von solchen Sprachmodellen im Gesundheitswesen für gefährlich.
6. Ich halte den Einsatz von solchen Sprachmodelle in der Psychiatrie für sinnvoll.
7. Ich halte den Einsatz von Sprachmodelle wie ChatGPT in der Psychiatrie für gefährlich.
8. Der Einsatz von solchen Sprachmodellen sollte in der Zukunft für die Psychiatrie erwogen werden.
9. Ein Beratungs-Chatbot auf Basis eines solchen Sprachmodells würde eine einfache Möglichkeit der niederschwelligen Kontaktaufnahme zur Psychiatrie im Bedarfsfall darstellen.
10. Der Einsatz von Sprachmodellen wie ChatGPT wäre hinsichtlich des Datenschutzes bedenklich.
11. Der Einsatz von solchen Sprachmodellen wäre ein zeitgemässes Hilfsmittel beim Management psychiatrischer Krisensituationen.
12. Ich denke, dass sich der Einsatz von solchen Sprachmodellen im Gesundheitswesen sich negativ auf die Patient:Innenversorgung auswirken wird.

**Chat GPT Studie Kasuistiken**

***Anpassungsstörung***

Nachdem meine langjährige Beziehung zu Ende ging, begann ich, mich unruhig, ängstlich und

nervös zu fühlen. Die Nächte wurden zu einer Herausforderung, da ich Schwierigkeiten hatte

einzuschlafen und durchzuschlafen. Jeder kleine Vorfall schien mich reizbar zu machen und

meine Konzentration litt darunter. Die täglichen Anforderungen überwältigten mich, und ich hatte das Gefühl, dass ich damit nicht umgehen konnte.

***Psychose***

Ich habe das Gefühl, Gefangener im Zentrum einer weit verbreiteten Verschwörung zu sein. Der

Verfolgungswahn und das Hören von Stimmen verstärken meine Ängste und lassen mich

glauben, dass ich allein im Kampf gegen diese Bedrohung stehe. Es gibt genug Beweise dafür,

aber niemand will mir glauben! Aus diesem Grund fühle ich mich emotionslos und antriebslos.

Meine Sorgen werden von anderen nicht verstanden. Ich habe kaum Zeit für soziales

Engagement oder persönliche Beziehungen, weil ich mich so sehr um diese Dinge kümmere.

***Depression***

Trotz einer stabilen Familie und gesunden Finanzen fühle ich eine anhaltende

Niedergeschlagenheit, verliere das Interesse an früheren Aktivitäten. Selbst bei geringer

körperlicher Anstrengung fühle ich einen starken Energieverlust und meine Konzentration lässt

nach. Aktivitäten, die ich einst genossen habe, bereiten mir keine Freude mehr, und mein

Interesse daran ist verloren gegangen. Dieser Zustand stellt mich vor ein Rätsel und ich fühle

mich zunehmend hilflos.
